# Prmt5: a guardian of the germline protects future generations

**DOI:** 10.15252/embj.201591054

**Published:** 2015-02-16

**Authors:** Rebecca V Berrens, Wolf Reik

**Affiliations:** 1 Epigenetics Programme, The Babraham InstituteCambridge, UK; 2 The Wellcome Trust Sanger InstituteCambridge, UK

## Abstract

Primordial germ cells (PGCs) are the embryonic precursors of the germ cell lineage that form sperm and egg cells. It is of great importance to preserve the germline from DNA damage and potentially from epimutations in order to ensure the survival of future generations. Recent research highlights the role of the protein arginine methyltransferase 5 (PRMT5) as an important player in DNA protection during germline development in the mouse (Kim *et al*, 2014 & Li *et al*, 2015).

See also: **S Kim *et al*** (November 2014) and **Z Li *et al*** (March 2015)

In early embryonic development shortly after implantation, a small group of cells emerge that develop into the precursors of the germ cells and are called primordial germ cells (PGCs). It is only this small group of cells that, in the proximal epiblast, exhibit the competence for PGC fate acquisition through the induction of the BMP network, which acts through SMAD signalling. The key players driving PGC specification are BLIMP1, PRDM14 and AP2γ (Magnúsdóttir *et al*, 2013). Germ line development starts with specification of PGCs (Phase I) during embryonic day (E) E6.5–7.25 of mouse development. From E7.5 onwards, PGCs migrate towards the developing gonad (Phase II), which they reach around E10.5 and undergo global demethylation of DNA and erasure of the repressive histone H3 lysine 9 dimethyl mark (H3K9me2) (Seki *et al*, 2007; Hajkova *et al*, 2008; Seisenberger *et al*, 2012).

PRMT5 catalyses the repressive histone mark H2A/H4R3me2 and arginine methyl-ation of nonhistone proteins such as spliceosomal Sm proteins (Meister *et al*, 2001; Bedford & Clarke, 2009; Vagin *et al*, 2009). As such, PRMT5 can work both on nuclear and on cytoplasmic targets. This protein arginine methyltransferase has also been suggested to be a key player of PGC specification or development. This is based on knockout (KO) experiments of the *Drosophila Prmt5* homologue (*dart5*), which leads to infertility (Anne *et al*, 2007). In the mouse, PRMT5 has been hypothesised to induce germline fate as an interactor of BLIMP1 (Ancelin *et al*, 2006).

A *Prmt5* KO in the mouse results in early embryonic lethality (Tee *et al*, 2010); therefore, the new investigations made use of conditional deletion of the gene during PGC formation. Kim *et al* (2014) found that PRMT5 is important for silencing particular transposon classes during global DNA de-methylation and in its absence, the DNA damage response is activated, while Li *et al* (2015) primarily report effects on RNA splicing through arginine methylation of Sm proteins and on PGC proliferation and survival. The studies are complementary in appearing to reveal multiple roles of PRMT5 in guarding genome integrity in PGCs.

In both studies, *Prmt5* KO led to male and female sterility through loss of germ cells at E9.5. However, neither of the studies found an effect of PRMT5 on PGC specification, as they could detect PGCs prior to E8.5 in the mutant embryos, nor did they find global demethylation of DNA or loss of H3K9me2 affected in *Prmt5* mutants. Interestingly, while PRMT5 was located in the cytoplasm during PGC specification (E6.5), it translocated into the nucleus at E8.0, prior to PGCs being affected by its loss, suggesting that nuclear functions of PRMT5 might be critical for the phenotypes observed. Both studies describe a PGC apoptotic phenotype, as well as changes in RNA splicing, confirming previous studies in which conditional deletion of *Prmt5* led to the same phenotype in neural progenitor cells through the methylation of Sm proteins (Bezzi *et al*, 2013). These phenotypes seem to reveal a more general role of PRMT5 rather than a germ cell-specific one.

Using immunofluorescence, Kim *et al* (2014) showed that the nuclear localisation of PRMT5 at E8.0 coincides with an enrichment of H2A/H4R3me2, which is lost again upon translocation of the arginine methyltransferase into the cytoplasm at E11.5. By ChIP, the researchers found PRMT5-dependent enrichment of H2A/H4R3me2 on active subclasses of LINE1 and IAP retrotransposons. Through RNAseq analysis of *Prmt5* KO PGCs, Kim *et al* (2014) showed an upregulation of DNA damage response genes as well as LINE1 and IAP transcripts. Thus, the authors suggest that PRMT5 is a keeper of DNA integrity during global epigenetic reprogramming which also affects transposons, at least in part. Consistent with this, they found expression of IAPs (but not LINE1s) in *Prmt5* knockout preimplantation embryos.

Once PRMT5 returns to the cytoplasm in PGCs, from E11.5, it has another function in methylating PIWI proteins, which have a role in piRNA-mediated *de novo* DNA methylation of transposons slightly later in germ cell development (Vagin *et al*, 2009). Hence, PRMT5 seems to be important both for retaining repression of some transposons when they first become demethylated and for their *de novo* methylation at the end of the demethylation phase.

Li *et al* (2015) hypothesised that PRMT5 may control the DNA damage response through alternative RNA splicing in PGCs by the methylation of Sm spliceosomal proteins. They carried out paired-end RNAseq analysis to call transcript splicing events in pluripotent ESCs, finding differentially spliced mRNAs in *Prmt5* mutant ESCs. Interestingly, the differentially spliced mRNAs were enriched for those involved in functions such as DNA damage response as well as RNA splicing, indicating that the genes affected by RNA splicing control splicing events themselves. The researchers confirmed that some of the alternatively spliced genes were also affected in mutant PGCs. Li *et al* (2015) conclude that PRMT5 potentially regulates the protein repertoire in developing PGCs by regulating RNA splicing.

The proposed roles for PRMT5 in PGC development are not mutually exclusive. For example, one could imagine a cause and effect role, in which the activation of transposable elements would lead to DNA damage and the DNA damage response is coordinated through PRMT5-dependent splicing regulation. Alternatively or additionally, DNA damage response activation may lead to apoptosis, thus eliminating germ cells in which transposon mobilisation occurred. This would protect the germline from DNA damage by stringent regulatory mechanisms, which may be interconnected. Further analyses of these intriguing effects of PRMT5 by more comprehensive epigenomic studies in PGCs will no doubt reveal its nuclear targets more fully. Perhaps it will also be interesting to look directly for transposon mobilisation in the mutant PGCs. A comprehensive understanding of the cytoplasmic and nuclear targets of PRMT5 together with mechanistic insights into the regulation of its localisation should provide further fascinating insights into this guardian of germline integrity.

**Figure 1:**
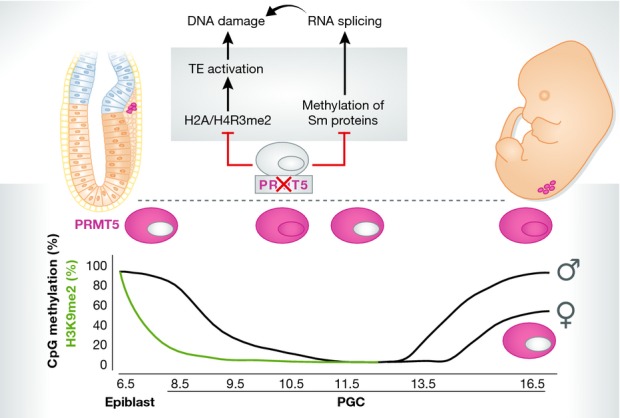
Conditional deletion of *Prmt5* in the mouse germline reveals the arginine methyltransferase as a guardian of germline integrity PRMT5 is required for survival and proliferation of primordial germ cells (pink) that is compromised between E10.5 and E13.5 in *Prmt5* KO PGCs. PRMT5 is dynamically expressed during mouse germline development. *Prmt5* conditional KO in the germline leads to reduced H2A/H4R3 methylation that induces activation of TEs and may subsequently lead to DNA damage. Additionally, lack of methylation of spliceosomal proteins leads to differential splicing events, which in turn control DNA damage response activation. This DNA damage may lead to apoptosis of PGCs with TE activity in order to protect the germline for future generations. Model based on Kim *et al* (2014) and Li *et al* (2015).
